# Early diagnosis of Alzheimer’s Disease based on multi-attention mechanism

**DOI:** 10.1371/journal.pone.0310966

**Published:** 2024-09-24

**Authors:** Xinli Yang, Kefen Hong, Denghui Zhang, Ke Wang

**Affiliations:** 1 College of Information Technology, Zhejiang Shuren University, Hangzhou, Zhejiang, China; 2 College of Information Engineering, Huzhou University, Huzhou, Zhejiang, China; Prince Sultan University, SAUDI ARABIA

## Abstract

Alzheimer’s Disease is a neurodegenerative disorder, and one of its common and prominent early symptoms is language impairment. Therefore, early diagnosis of Alzheimer’s Disease through speech and text information is of significant importance. However, the multimodal data is often complex and inconsistent, which leads to inadequate feature extraction. To address the problem, We propose a model for early diagnosis of Alzheimer’s Disease based on multimodal attention(EDAMM). Specifically, we first evaluate and select three optimal feature extraction methods, Wav2Vec2.0, TF-IDF and Word2Vec, to extract acoustic and linguistic features. Next, by leveraging self-attention mechanism and cross-modal attention mechanisms, we generate fused features to enhance and capture the inter-modal correlation information. Finally, we concatenate the multimodal features into a composite feature vector and employ a Neural Network(NN) classifier to diagnose Alzheimer’s Disease. To evaluate EDAMM, we perform experiments on two public datasets, i.e., NCMMSC2021 and ADReSSo. The results show that EDAMM improves the performance of Alzheimer’s Disease diagnosis over state-of-the-art baseline approaches on both datasets.

## Introduction

Alzheimer’s Disease (AD), as a primary type of dementia in the elderly, is currently incurable. This disease primarily occurs in the elderly aged between 65 and 90 years, characterized by a gradual onset of memory decline. As the condition progresses, patients can suffer from memory loss and neurological functional abnormalities such as a decline in language ability and cognitive function. These neurological functional abnormalities have a significant impact on the daily life of the patients [1]. Researches have revealed that the conversion rate from Mild Cognitive Impairment (MCI) to AD is exceptionally high, and the number of AD patients is increasing annually [2]. herefore, detecting the disease in its early stages is crucial [3]. Early diagnosis and intervention can effectively slow down the progression of the disease.

In order to achieve early diagnosis of Alzheimer’s Disease, various methods can be employed, including artificial intelligence, cerebrospinal fluid biomarker diagnosis [4] and imaging techniques [5]. Particularly in the field of imaging technology, significant advancements have been made. Ebrahimi et al. [6] employed the ResNet-18 neural network along with the Temporal Convolutional Network (TCN) and various types of Recurrent Neural Networks as sequence models to process Magnetic Resonance Imaging (MRI) images. This approach achieves good performance for Alzheimer’s Disease diagnosis since it can better understand temporal dependencies between MRI slices. Houria et al. [7] developed a novel multimodal MRI fusion strategy to detect white matter changes and cerebral gray matter atrophy in AD patients, and achieved even better performance.

However, imaging techniques are often invasive and expensive. Therefore, the search for a non-invasive and cost-effective diagnosis method is crucial. Currently, within the non-invasive domain, the most prominent features of the early stages of Alzheimer’s Disease are language features, such as non-fluent speech, difficulty in word retrieval, and repetitive speech. Previous studies have indicated that the language centers in AD patients are damaged, affecting their abilities in thought expression, understanding others’ speech, and memory of vocabulary [8, 9]. There are two kinds of language features for early diagnosis of Alzheimer’s Disease, i.e., acoustic features and linguistic features.

Some researchers have focused on acoustic features to diagnose Alzheimer’s Disease. Qin et al. [10] used the Wav2Vec2.0 for audio feature extraction, achieving significant results in AD diagnosis tasks. Liu et al. [11] created a new audio dataset comprising AD patients and Healthy Controls(HC) groups, and employed the Logistic Regression Cross-Validation model for classification, demonstrating the effectiveness of extracting spectrogram features from audio data. Calzà et al. [12] utilized Support Vector Machine (SVM) and RF algorithms to analyze audio samples, distinguishing between HC groups and those with MCI. Wang et al. [13] analyzed the percentage of silence duration (PSD), finding a notable increase in PSD in patients with MCI and AD, confirming it as a reliable diagnostic biomarker for early AD and MCI. Agbavor et al. [14] used pretrained audio models to extract features from patient audio and evaluated them on ADReSSo.

Some researchers have focused on linguistic features to diagnose Alzheimer’s Disease. For instance, Adhikari et al. [15], targeting the Nepali language, employed techniques such as TF-IDF, CountVectorizer (CV), Word2Vec, and FastText to transform text documents into vector representations. In terms of machine learning models, the Naive Bayes classifier demonstrated optimal performance when utilizing CV and TF-IDF vectorization techniques. Regarding deep learning models, the best results were achieved with a Convolutional Neural Network (CNN) model incorporating Word2Vec when applying attention mechanisms. Ilias et al. [16] used multi-task learning methods and transformer-based models for AD diagnosis, showing high accuracy in both single-task and multi-task learning scenarios, and revealing language differences between AD and non-Alzheimer’s Disease (Non-AD) patients. Khan et al. [17] employed traditional machine learning models, sequential deep learning models, as well as XLNet and BERT models for the diagnosis of AD. These models demonstrated varying levels of accuracy in the automatic diagnosis of cognitive memory loss language indicators in AD patients. In later work, they [18] utilized the Bag-of-Words method for feature extraction and developed a Stacked Deep Dense Neural Network model for text classification. Concurrently, they employed audio recording data for the diagnosis of AD.

Previous researches have indicated that single-modal methods may not be adequate for early diagnosis of Alzheimer’s Disease since lack of information. Huang et al. [19] mathematically validated the superiority of multimodal methods over any single-modal model. Therefore, multimodal methods are emerging. By integrating data from different sources, multimodal methods can generate more accurate semantic representations and improve the overall performance of models, overcoming limitations of single-modal methods. Ying et al. [20] identified AD using multimodal features encompassing acoustic and language features. They extracted traditional acoustic features using the IS10 toolkit, deep acoustic features with a fine-tuned Wav2Vec2.0 model, and linguistic features with a fine-tuned BERT model. After concatenating these features, classification was performed using an SVM classifier. This method achieved accuracies of 89.1% for long audio and 84.0% for short audio. However, the method simply concatenates features without fully considering the correlations between modalities. Mittal et al. [21] also proposed a multimodal deep learning method for simultaneous diagnosis of Alzheimer’s Disease using both speech and corresponding transcripts. This method employed transfer learning to address data scarcity issues and tested the feasibility of using text generated by an Automatic Speech Recognition (ASR) system instead of manual transcripts. It achieved an accuracy of 85.3% on the DementiaBank dataset and conducted an analysis of the model’s age and gender biases. Pan et al. [22] employed an ASR system to obtain time alignment information and confidence scores for audio segments, enhancing the robustness of acoustic feature extraction. They integrated acoustic and linguistic features, utilizing Bidirectional Long Short-Term Memory and attention mechanisms to classify the combined features. The results indicated that this method could accurately discriminate audio segments containing information related to cognitive impairments, providing robust support for early diagnosis. Li et al. [23] proposed several effective methods for extracting clues more relevant to Alzheimer’s Disease from advanced acoustic and linguistic features. The validation was conducted by comparing the performance of combinations of acoustic, language, and task-related features. Martinc et al. [24] conducted an analysis of Alzheimer’s Disease diagnosis methods using audio feature engineering. They utilized the OpenSmile toolkit and GloVe to extract acoustic and linguistic features from speech segments, subsequently constructing Active Data Representation features.

In summary, domestic and international researches have indicated significant progress in the early diagnosis of Alzheimer’s Disease through multimodal methods. However, many challenges, such as the extraction of adequate features and the integration of diverse features, still exist. In response to the aforementioned issues, this study has made the following key contributions:

1. We conducted a comprehensive analysis and comparison of common feature extraction techniques, and found an optimal combination of three techniques (Wav2Vec2.0, TF-IDF and Word2Vec) for Alzheimer’s Disease diagnosis.
2. We propose a novel diagnosis model EDAMM for Alzheimer’s Disease. EDAMM leverages a novel feature fusion strategy based on multi-attention mechanisms, including self-attention mechanism and cross-modal attention mechanism.
3. We compare EDAMM with three state-of-the-art baseline approaches on a Chinese dataset NCMMSC2021 and seven state-of-the-art baseline approaches on an English dataset ADReSSo, respectively. The experiment results show that EDAMM achieves better performance over the baselines for Alzheimer’s Disease diagnosis.

## EDAMM

We name the model EDAMM, which stands for ‘Early Diagnosis of Alzheimer’s Disease based on Multimodal Attention’. Here, ‘E’ stands for ‘Early’, ‘D’ for ‘Diagnosis’, ‘A’ for ‘Alzheimer’s Disease’, the first ‘M’ for ‘Multimodal Attention’, and the second ‘M’ for ‘Model’.

**Fig 1:**
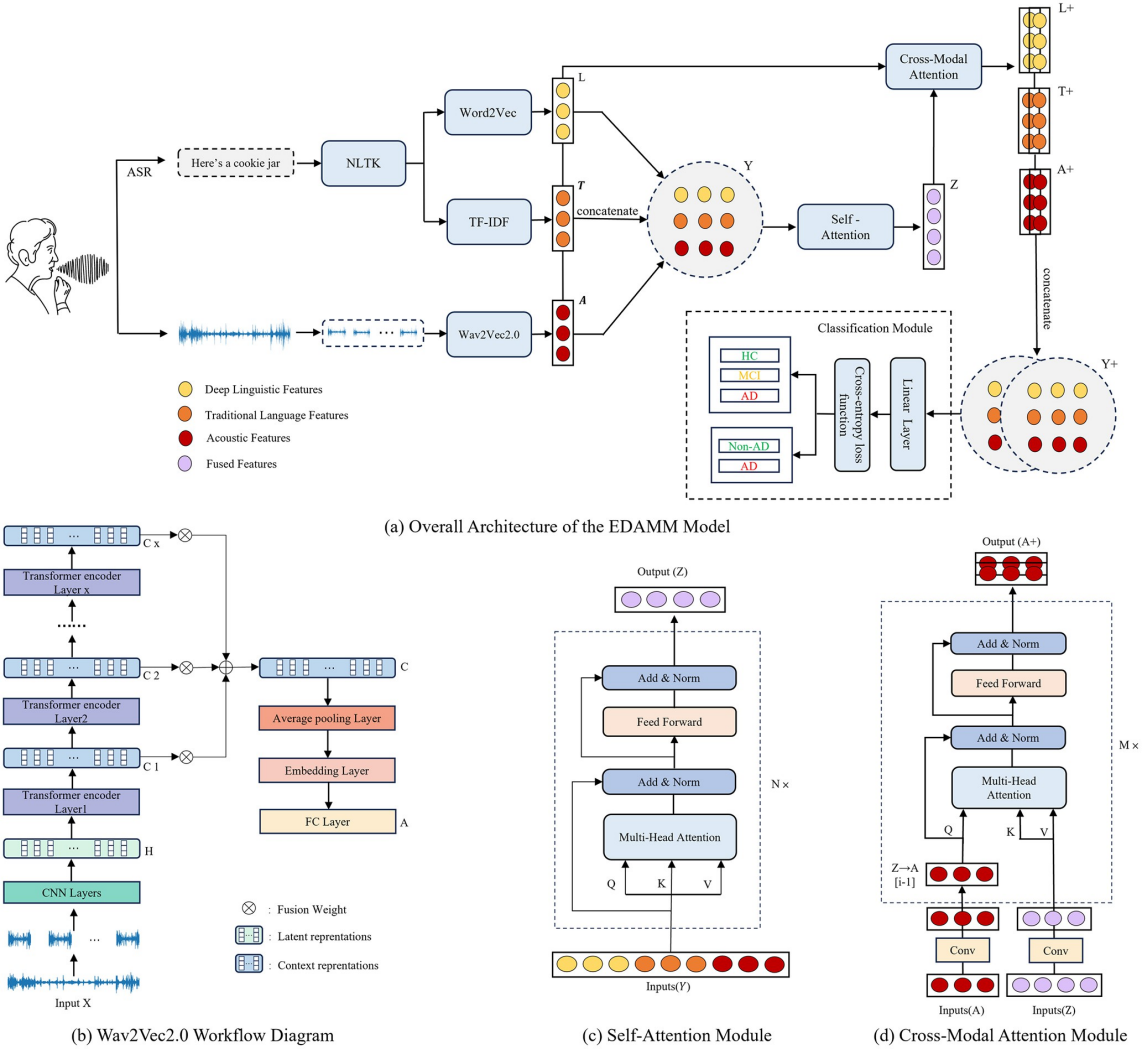
Overall architecture and modules of EDAMM.

In EDAMM, we first use three feature extraction methods, i.e., Wav2Vec2.0, TF-IDF and Word2Vec, to extract acoustic and linguistic features. Subsequently, the extracted features from these three methods are concatenated to form a fused multimodal feature sequence *Y*. Through self-attention mechanism, further feature extraction is performed on *Y*, integrating semantic information to obtain the fused feature *Z*. Following this, cross-modal attention mechanisms are utilized, allowing the vectors from the three modalities to access information within the fused feature, resulting in three enhanced features:*L*+, *T*+ and *A*+. Finally, the three enhanced features are concatenated to create the ultimate feature *Y*+, which is used for training using Neural Network(NN) classifier. The overall architecture of this model is shown in Fig 1(a).

### Acoustic features based on Wav2Vec2.0

Wav2Vec2.0 [25] is an acoustic feature extraction method that consists of multiple CNNs, vector quantization modules, and Transformer-based context encoding. This technique can extract meaningful sound representations from audio signals to process sound data more efficiently in subsequent tasks.

In our study, the process of Wav2Vec 2.0 are illustrated in Fig 1(b). Firstly, the initial audio input X are uniformly divided into several audio segments. Specifically, in ADReSSo the initial audio is divided by 10 seconds, while in NCMMSC2021, the initial audio is divided by 20 seconds for long speech and 6 seconds for short speech. Note that the segmentation lengths vary due to the language differences of the two datasets, and the segmentation lengths are the same in the TF-IDF and Word2Vec methods.

Next, these fixed-length segments are encoded through multiple layers of CNNs, generating latent audio representations *H* The hidden layer in each CNN layer includes batch normalization and ReLU activation function to enhance the nonlinear relationship and generalization ability of model learning. After each CNN layer, there are dropout layers for randomly discarding the outputs of a subset of neurons, which helps to prevent overfitting and ensures that the model performs well on different datasets.

Third, the latent audio representation *H* is masked and fed into a Transformer-based context network. After weighted fusion of the outputs of each Transformer block, a context feature sequence *C* is generated. Each Transformer block includes a self-attention layer and a feedforward network, interspersed with a normalization layer and a dropout layer, which enhances the model’s ability to capture long-distance dependencies.

Subsequently, the audio representations processed by the Transformer are further passed through an average pooling layer, compressing the long sequence into a fixed-size 1024-dimensional acoustic feature vector. This step aims to simplify information and reduce the complexity of subsequent processing. Then, an embedding layer with a ReLU activation function further transforms the 1024-dimensional feature vector into a 192-dimensional feature vector to further extract key information from the audio signal.

Finally, these features undergo a linear transformation through a fully connected layer, producing feature representations *A* that are suitable for various tasks. This architecture optimizes the transformation process from raw audio to useful acoustic features, emphasizing the ability to capture long-range dependencies, which is crucial for understanding the context and dynamic changes in speech.

Compared with other feature extraction techniques, Wav2Vec 2.0 focuses more on extracting acoustic properties such as waveforms and spectra rather than directly parsing linguistic content. This approach helps maintain the consistency of features, unaffected by language variations and data complexity. Additionally, it does not require large amounts of labeled data, making it more suitable for extracting acoustic features in multimodal datasets.

### Linguistic features based on TF-IDF

To obtain multimodal data, we extract linguistic features from the text. We convert the segmented speech into text using the IFLYTEK API(https://www.xfyun.cn’services/lfasr), a speech-to-text tool. Then, we employ the TF-IDF algorithm to extract features from these texts. The TF-IDF algorithm weights words effectively based on Their Frequency (TF) in a document and their Inverse Document Frequency (IDF) across all documents, highlighting key information in the text. This method efficiently identifies and emphasizes important information in the text while naturally disregarding high-frequency but less informative stopwords.

In our study, we use the Natural Language Toolkit (NLTK) to preprocess the text, which includes text tokenization and the removal of stopwords. We set the maximum number of features at 192, meaning that each document is transformed into a vector containing 192 elements, each representing the TF-IDF score of a specific word. By this method, we calculate the TF value of each word in a specific document as well as its IDF value. The TF-IDF value for each word is obtained by multiplying its TF value by its IDF value, resulting in the feature vector *T*. The entire process is described in the following formula.

$$\begin{array}{c} TF(w,d)=\frac{N^{w,d}}{N^{d}} \end{array}$$ (1)

$$\begin{array}{c} IDF\left(w,D\right)=\text{log}\left(\frac{N_{D}}{n_{w}+1}\right) \end{array}$$ (2)

$$\begin{array}{c} TF-IDF(w,d,D)=TF(w,d)\times IDF(w,D) \end{array}$$ (3)

$$\begin{array}{c} T=(TF-IDF\left(w_{1},d,D\right),TF-IDF(w_{2},d,\\ D),\ldots ,TF-IDF\left(w_{n},d,D\right))\times IDF\left(w,D\right) \end{array}$$ (4)

Here, *w* represents a word, *d* represents a document, *N*_*w*,*d*_ represents the number of times the word *w* appears in document *d*, *N*_*d*_ represents the total number of words in document *d*. *D* represents the document set, *n*_*w*_ represents the number of documents containing the word *w*, and *N*_*D*_ represents the total number of documents. *w*_1_, *w*_2_, …, *w*_*n*_ represents the words in the document.

Compared with other feature extraction techniques, TF-IDF is able to capture unique linguistic patterns, such as repetitive use, grammatical errors, and confused words, which are more prevalent in the speech of AD patients.

### Linguistic features based on Word2Vec

Word2Vec, proposed by Mikolov et al. [26], is a model widely used in natural language processing. It efficiently maps words to continuous vector spaces, capturing semantic relationships. The vector representation produced by Word2Vec preserves semantic information among words, allowing similar words to be closer in vector space. Through dimensionality reduction, Word2Vec transforms originally sparse high-dimensional word vectors into low-dimensional dense vectors, thereby enhancing model training and storage efficiency.

The Word2Vec model primarily has two training algorithms: Skip-gram and Continuous Bag Of Words (CBOW). In our study, the segmented speech is also converted to text through the IFLYTEK API. Then CBOW is adopted, which maximizes the probability of predicting the central word given the context words through its objective function. The training objective function of CBOW is as follows:

$$\begin{array}{c} logP(w|c) \end{array}$$ (5)

Here, *logP*(*w*|*c*) represents the probability of the word occurring given the context words *c*. Taking the negative logarithm and summing this probability yields the conditional entropy, which measures the average information content under the given condition. We train the Word2Vec model using the preprocessed text data, forming the feature vector *L*.

Compared with other feature extraction techniques, Word2Vec is able to capture the semantic relationship between words and helps to identify deep patterns in language usage. The joint application of Word2Vec and TF-IDF can better capture semantic language patterns and provide strong support for early diagnosis of Alzheimer’s Disease.

### Self-attention module

Self-attention module is constructed using Transformer encoder based structure [27], which mainly consists of multi-head attention mechanism and feedforward fully connected layer. The structure has a high degree of parallel computing power and is able to focus on all positions in the sequence at the same time, especially when dealing with long sequences. The multi-head attention mechanism has multiple heads, each focused on learning useful information about a particular modal. Therefore, the multi-head attention mechanism can selectively focus on key features of a particular modal, and better learn associations between different modal features.

In our study, self-attention module transforms concatenated vector *Y*(consisting of *A*,*T* and *L*) into *Z* through multiple hidden layers, as shown in Fig 1(c). First, *Y* is divided into several parts according to its dimension *d*, each part is a head, with a total of *h*. Each head undergoes a linear transformation through its corresponding hidden layer. The attention matrix of the head *i* is calculated as follows:

$$\begin{array}{c} head_{i}=softmax(\frac{Q_{i}{\left(K_{i}\right)}^{T}}{\sqrt{d/h}})V_{i} \end{array}$$ (6)

Here, $Q_{i}=W_{i}^{Q}Y$, $K_{i}=W_{i}^{K}Y$ and $V_{i}=W_{i}^{V}Y$ respectively represent query vector, key vector and value vector after linear change. $W_{i}^{Q}$,$W_{i}^{K}$,$W_{i}^{V}$ is the weight of three random initialization matrix.

Then, the attention matrix *head*_1_, *head*_2_, …, *head*_*h*_ obtained by all heads is spliced, and the spliced result is multiplied with the weight matrix *W*^*o*^ of a hidden layer to obtain the final output of multi-head attention *F*. This hidden layer can further integrate information from different heads to enhance the model’s understanding of contextual information. The formula is calculated as follows:

$$\begin{array}{c} F=Concat\left(head_{1},head_{2},\ldots ,head_{h}\right)W^{o} \end{array}$$ (7)

In order to prevent overfitting and improve the generalization ability of the model, a dropout layer is introduced to randomly discard part of the attention weights. Finally, the multi-modal features are fused by residual connection and layer normalization, and the context information fusion feature *Z* is obtained by converting the output *F* of multi-attention through the full connection layer. The details are as follows:

$$\begin{array}{c} \begin{array}{c} Z=LN(LN\left(Y+F;\gamma_{LN1},\beta_{LN1}\right)+ReLU(LN\left(Y+F;\gamma_{LN1},\beta_{LN1}\right)\\ \cdot W_{1FFN})\cdot W_{2FFN};\gamma_{LN2},\beta_{LN2}) \end{array} \end{array}$$ (8)

Here, *LN* is the layer normalization and *Relu* is the activation function. *γ*_*LN*1_, *γ*_*LN*2_ and *β*_*LN*1_, *β*_*LN*2_ are parameters that respectively control the normalization scaling and offset, each applied to two separate layer normalizations. *W*_1*FFN*_ and *W*_2*FFN*_ represent the weight matrices for linear transformations. The former transforms the output *F* of the multi-head attention to an intermediate layer with higher dimensions, while the latter transforms the output of the intermediate layer to the final output dimensions.

### Cross-modal attention module

The cross-modal attention module is constructed based on Crossmodal Transformer model structure [28]. The design fully allows the information interaction between different modals, significantly enhances the representation ability of each modal, and utilizes the correlation and complementarity between modals to promote the fusion between modal. The cross-modal attention module is similar to the self-attention module except that the query vector (*Q*), the key vector (*K*) and value vector (*V*) are derived differently.

Fig 1(d) shows an example how feature vector *A* is transformed to *A*+ with *Z* based on the cross-modal attention module in our study. First, we use a one-dimensional convolution layer to unify the dimensions of the input vectors *A* and *Z*. The unified dimension is denoted as *d*_*cross*_. Similarly, in the cross-modal multi-head attention layer, each head undergoes a linear transformation through its corresponding hidden layer. The attention matrix of the head *i* is calculated as follows:

$$\begin{array}{c} head_{i}^{cross}=softmax(\frac{Q_{i}^{A}{\left(K_{i}^{Z}\right)}^{T}}{\sqrt{d_{cross}/h_{cross}}})V_{i}^{Z} \end{array}$$ (9)

Here, $Q_{i}^{A}=U^{A}W_{i}^{Q^{cross}}$ and $K_{i}^{Z}=ZW_{i}^{K_{cross}}$,$V_{i}^{Z}=ZW_{i}^{V_{cross}}$ respectively represent the results of linear transformations applied to the acoustic modality *A*_*cross*_ and the fused feature *Z*_*cross*_. $W_{i}^{Q^{cross}}$, $W_{i}^{K^{cross}}$ and $W_{i}^{V^{cross}}$ are the weight matrices for linear transformations corresponding to the I-th attention head of the respective modalities. *h*_*cross*_ indicates the number of attention heads.

Then, the attention matrices $head_{1}^{cross},head_{2}^{cross},\ldots ,head_{h}^{cross}$ obtained by all heads is spliced, and the spliced result is multiplied with the weight matrix $W_{cross}^{o}$ of a hidden layer to obtain the output of multi-head attention *F*_*cross*_. The formula is as follows:

$$\begin{array}{c} F_{cross}=Concat(head_{1}^{cross},head_{2}^{cross},\ldots ,head_{h2}^{cross})W_{cross}^{o} \end{array}$$ (10)

Similarly, in order to enhance the generalization ability of the model, a dropout layer is introduced to randomly discard part of the attention weights. Finally, the output is computed using residual connections, layer normalization, and a fully connected layer, resulting in *A*+.

The self-attention module and the cross-modal attention module are crucial to the multimodal approach. They can fully consider the potential association between speech and text data. Therefore, they help a lot in the early diagnosis of AD.

### Classification module

We compare various classifiers, and choose the most effective one—the NN classifier. The NN classifier includes a linear layer and uses the cross-entropy loss function for classification. Specifically, the linear layer maps the input features to scores for different categories. The Cross-Entropy Loss function internally utilizes a softmax operation to convert the scores from the Linear Layer into a probability distribution, ensuring that each category’s probability is within the [0, 1] range and the total sum of probabilities for all categories equals 1. By calculating the loss, the model learns to better classify input data, enabling effective classification.

## Experimental results and analysis

### Datasets

We conduct experiments using two widely used datasets: ADReSSo [29] and NCMMSC2021 [30].

#### NCMMSC2021

NCMMSC2021 is a Chinese speech dataset used for three classification, including cases of AD, MCI and HC patients. It has two types of audio data: long speech (30-60 seconds) and short speech (6 seconds). The short speech is segmented from the long speech. We have experimented with different segmentation lengths (5-10 seconds) and found that short speeches with 6 seconds are the best in diagnosis performance. In addition, all kinds of short speeches are less accurate than long speeches because they are truncated from the long speeches, which results in information loss. Therefore, we just choose short speeches with 6 seconds as an example for experimental illustration in our experiment. To evaluate the robustness of the model, the dataset is divided into training and testing groups. The long speech training group contains 280 audio samples, and the test group contains 119 audio samples. The short speech training group included 4699 audio samples and the test group included 1153 audio samples.

**Fig 2:**
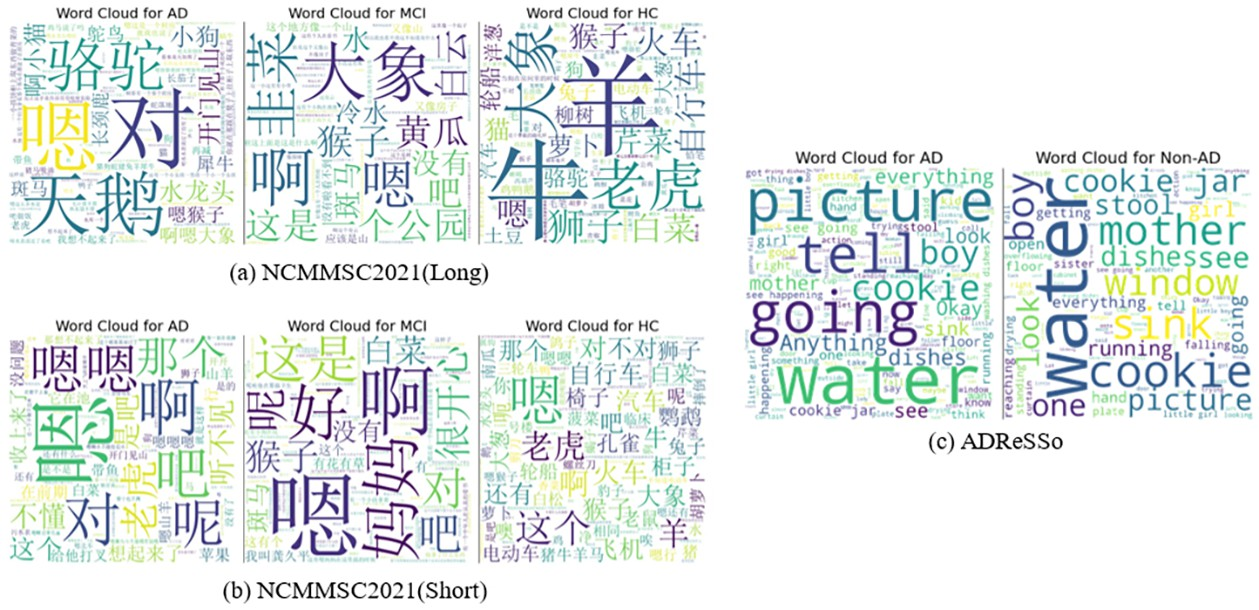
Word Clouds of NCMMSC2021 and ADReSSo.

To analyze NCMMSC2021, word cloud visualizations were used, as shown in Fig 2(a) and 2(b). Observations of the word clouds revealed a higher occurrence of modal words, such as ‘um’ and ‘yes’, in the language expressions of AD patients. In contrast, the word cloud for the HC group displayed a richer and more varied vocabulary usage. This finding provides a significant perspective and data support for further studies on the linguistic features of AD patients.

#### ADReSSo

ADReSSo is an English speech dataset used for binary classification, including cases of AD and Non-AD patients. It comprises 166 audio samples, with 87 from AD patients and 79 from Non-AD participants, varying in length from 22 to 235 seconds.

ADReSSo is derived from the picture description task of the Boston Diagnostic Aphasia Examination, which requires participants to describe a ‘Cookie Theft’ picture and record their descriptions as audio, forming the foundation of ADReSSo. To minimize audio discrepancies caused by varying recording conditions, all audio files are subjected to uniform noise reduction and volume normalization procedures. Similar with NCMMSC2021, word cloud visualizations were employed to analyze ADReSSo, as illustrated in Fig 2(c).

### Experimental setup

#### Experimental requirements

*Hardware*. A server equipped with an NVIDIA GeForce RTX 2060 SUPER.

*Software*. Python 3.8.19, using PyTorch 1.12.1 as the main machine learning framework.

Table 1 shows the detailed experimental requirements for the datasets in our study:

**Table 1:** Experimental requirements.

Dataset	Running time(seconds)	Storage requirements(GB)	GPU requirements(GB)
NCMMSC2021(long)	13.18	0.66	0.15
NCMMSC2021(short)	32.59	0.65	0.6
ADReSSo	65.83	1.63	1.6

#### Parameter settings

In the experimental section, AdamW is used as the optimizer, with a linear warm-up for learning rate adjustments. Table 2 displays the parameter settings in our study. These parameters include settings for the Wav2Vec2.0 model, Word2Vec model, TF-IDF algorithm, self-attention, and cross-modal attention mechanisms. Note that we use the same parameter settings for both NCMMSC 2021 and ADReSSo datasets to guarantee the robustness of EDAMM.

**Table 2:** Parameters settings.

Model	parameters	Dataset
Wav2Vec2.0	Epochs	32
	Learning rate	1e-5
	batch size	8
Word2Vec	Learning rate	0.025
	Epochs	5
	Window size(Number of samples)	5
	Word vector dimension	100
	Workers	4
	Minimum frequency	1
TF-IDF	Word vector dimension	192
Self-attention	Epochs	100
	Dropout	0.1
	Number of encoder layers	2
	Attention head	11
Cross-modal attention	Epochs	100
	Dropout	0.1
	Number of encoder layers	1
	Attention head	2

#### Evaluation metrics

We use four common evaluation metrics, i.e., Accuracy, Precision, Recall and F1, to evaluate the effectiveness of EDAMM. These metrics are derived by a confusion matrix. For ADReSSo as an example, *TP* denotes the number of samples correctly diagnosed as AD and *TN* denotes the number of samples correctly diagnosed as Non-AD. *FP* denotes the number of samples that are incorrectly diagnosed as AD, and *FN* denotes the number of samples that are incorrectly diagnosed as Non-AD. The specific formulas for calculating these evaluation metrics are as follows:

$$\begin{array}{c} Accuracy=\frac{TP+TN}{TP+TN+FP+FN} \end{array}$$ (11)

$$\begin{array}{c} Precision=\frac{TP}{TP+FP} \end{array}$$ (12)

$$\begin{array}{c} Recall=\frac{TP}{TP+FN} \end{array}$$ (13)

$$\begin{array}{c} F1=\frac{2\times Precision\times Recall}{Precision+Recall} \end{array}$$ (14)

Note that due to limited dataset size, we take the following three methods to avoid potential overfitting and make the experiment results more convincing. First, we increase the sample size of the dataset by segmenting the initial audio into fragments. Second, we use 10-fold cross validation in all experiments. Third, we run 100 iterations out of random splits of the datasets and calculate the mean and standard deviation for each metric to improve the reliability of the results.

### Comparative experiments

#### Comparison of various combination of feature extraction methods

In the field of speech and text data feature extraction, researchers have been continuously exploring various combination methods to improve the classification performance for AD. Different combination can have a high impact on the classification performance for AD. To find the optimal combination of feature extraction methods, we try various combination of five popular feature extraction technologies, including Wav2Vec2.0, IS10, BERT, TF-IDF and Word2Vec, and conduct a comparative experiment on the NCMMSC2021 and ADReSSo datasets.

Table 3 displays the results of early AD diagnosis using various feature extraction combinations applied to the NCMMSC2021 and ADReSSo datasets.

**Table 3:** Results of different feature combinations in the dataset.

Dataset	Method	Accuracy(%)	Precision(%)	Recall(%)	F1(%)
NCMMSC2021(long)	IS10+BERT+TF-IDF	75.9±2.5	75.2±2.2	74.0±2.7	74.4±2.5
	Wav2Vec2.0+IS10+BERT	86.2±2.1	86.1±1.9	85.7±2.5	85.8±2.0
	Wav2Vec2.0+IS10+TF-IDF	86.4±1.9	86.2±2.0	85.9±2.0	85.2±2.5
	Wav2Vec2.0+TF-IDF+Word2Vec	90.2±1.5	90.2±1.3	89.9±1.7	90.5±1.4
NCMMSC2021(short)	IS10+BERT+TF-IDF	71.6±2.9	72.2±2.6	69.8±3.0	70.0±2.8
	Wav2Vec2.0+IS10+BERT	79.7±2.0	78.9±2.1	78.6±1.9	78.6±2.0
	Wav2Vec2.0+IS10+TF-IDF	81.2±1.3	80.0±1.4	79.8±1.5	79.9±1.3
	Wav2Vec2.0+TF-IDF+Word2Vec	83.2±2.1	82.7±2.0	82.5±2.5	82.7±2.5
ADReSSo	IS10+BERT+TF-IDF	83.5±3.0	85.1±2.7	83.6±3.0	83.2±3.1
	Wav2Vec2.0+IS10+BERT	86.7±1.4	87.0±2.0	86.6±1.6	86.5±1.5
	Wav2Vec2.0+IS10+TF-IDF	84.2±1.2	85.2±1.2	84.2±1.5	84.0±1.3
	Wav2Vec2.0+TF-IDF+Word2Vec	85.5±1.3	85.9±1.2	85.4±1.5	85.0±1.8

In NCMMSC2021, the combination of Wav2Vec2.0, TF-IDF and Word2Vec performs exceptionally well on both long and short speech samples, demonstrating its effectiveness and feasibility in the early diagnosis of Alzheimer’s Disease.

In ADReSSo, the Wav2Vec2.0+ IS10+BERT combination yielded the highest classification accuracy at 86.7%. It is important to note that BERT, being a comprehensive language model, demands extensive training data and computational resources for optimal performance. In our study, the constrained data scope underutilized BERT’s potential. Also, BERT’s fine-tuning relies on task-specific labeled data, which might be scarce or non-existent for languages used by AD and MCI patients or in dialects. Furthermore, BERT’s attention mechanism, oriented towards entire input sequences, does not effectively capture localized language patterns, semantic nuances, or the significance of specific words pertinent to AD and MCI patients.

In conclusion, this paper evaluates various feature extraction methods and finds an optimal combination of three techniques (Wav2Vec2.0, TF-IDF and Word2Vec). This combination demonstrates complementarity in capturing linguistic features and patterns, enabling a better understanding of language changes in AD patients.

#### Comparison of classification modules

A good classification module is crucial for AD diagnosis. Therefore, we make a comparison of four different classifiers, i.e., SVM, Random Forests (RF), Naive Bayes (NB) and NN classifier. By comparing different classifiers, we can evaluate the strengths and limitations of each classifier and choose the classifier that best suits for AD diagnosis.

As can be seen from the data in Table 4, NN classifier performs the best in most cases. SVM performs slightly better than NN classifier only in NCMMSC2021(short) dataset. In contrast, Naive Bayes (NB) and Random Forests (RF) are relatively poor and unstable in different datasets.

In summary, the NN classifier outperforms other classifiers in most cases and can be stable in different datasets. Therefore, we choose the NN classifier for AD diagnosis.

**Table 4:** Comparison results of different classifiers for AD diagnosis.

Dataset	classifier	Accuracy(%)	Precision(%)	Recall(%)	F1(%)
NCMMSC2021(long)	SVM	89.1	88.6	88.6	88.6
	RF	84.9	84.6	84.0	84.1
	NB	34.5	26.4	35.4	28.5
	NN	90.2	90.2	89.9	90.5
NCMMSC2021(short)	SVM	84.0	83.5	83.5	83.5
	RF	45.7	44.2	43.9	40.1
	NB	32.2	36.4	35.1	22.1
	NN	83.2	82.7	82.5	82.7
ADReSSo	SVM	82.3	82.6	82.3	82.2
	RF	72.9	73.5	72.7	72.4
	NB	68.5	58.4	68.0	61.4
	NN	85.5	85.9	85.4	85.0

#### EDAMM validity verification

To verify the effectiveness of EDAMM, we compare it with three state-of-the-art baseline approaches on the Chinese dataset NCMMSC2021 and seven state-of-the-art baseline approaches on the English dataset ADReSSo, respectively. The results are shown in Table 5. All the metrics are derived by calculating the average results of different groups.

**Table 5:** Results of Alzheimer’s Disease diagnosis by different methods.

Dataset	Method	Accuracy(%)	Precision(%)	Recall(%)	F1(%)
NCMMSC2021(long)	Official baseline	79.8	79.9	78.5	78.6
	Qin [10]	83.2	83.0	82.8	82.8
	Ying [20]	89.1	88.7	88.8	88.6
	**Ours**	**91.6**	**91.3**	**91.5**	**91.3**
NCMMSC2021(short)	Official baseline	74.0	72.3	73.7	71.8
	Qin [10]	78.0	76.9	76.5	76.2
	Ying [20]	84.0	83.6	83.5	83.5
	**Ours**	**85.2**	**84.6**	**84.6**	**84.6**
ADReSSo	Official baseline	78.9	/	/	/
	Chen [31]	81.9	/	/	82.6
	Zhu [32]	83.1	83.6	83.0	83.0
	Qiao [33]	83.1	83.5	83.5	83.0
	Pan [34]	84.5	84.7	84.6	84.5
	Wang [35]	77.2	78.7	74.0	76.3
	Ying [20]	83.7	83.8	83.8	83.8
	**Ours**	**86.7**	**87.0**	**86.7**	**86.6**

As demonstrated in Table 5, EDAMM achieves the best performance on both datasets. In long speech tasks, EDAMM exhibits an 11.8% improvement over the baseline, an 8.4% increase compared to Qin et al.’s method, and a 2.5% enhancement relative to Ying et al.’s method. In short speech tasks, EDAMM achieves an 11.2% improvement over the baseline, a 7.2% increase compared to Qin et al.’s method, and a 1.2% enhancement relative to Ying et al.’s method. It is noteworthy that Qin et al. exclusively utilized the Wav2Vec2.0 model for acoustic feature extraction from NCMMSC2021, without incorporating various feature fusion techniques. Although Ying et al. combined voice and text data, they only performed simple concatenation in feature fusion. In ADReSSo, there was also an improvement compared to all seven methods, with a 7.8% enhancement over the baseline.

**Fig 3:**
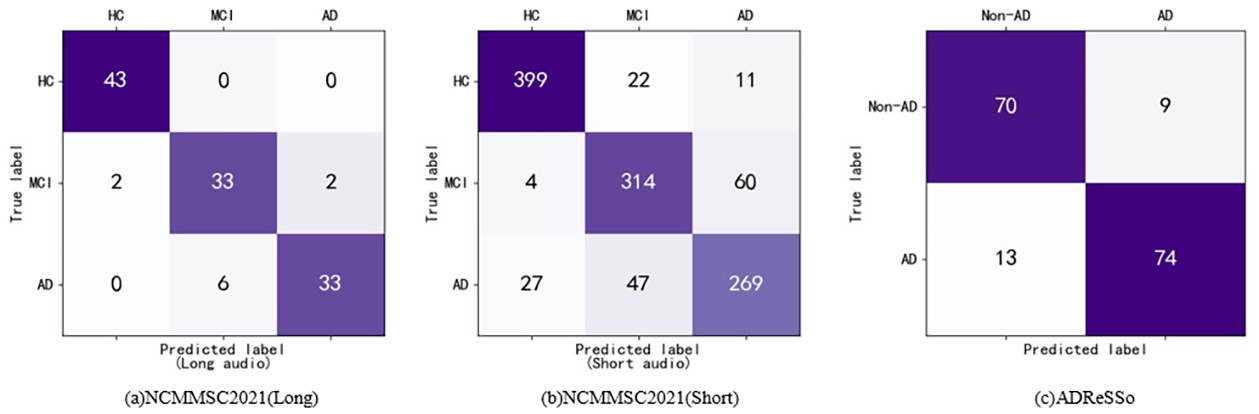
Confusion mtrices for NCMMSC2021 and ADReSSo datasets.

Note that in the study of early diagnosis of Alzheimer’s Disease, the evaluation metrics related to AD group are of greater significance. Generally, we can predict normal individuals with relatively high accuracy, while we may predict AD patients with lower accuracy. Therefore, the key to improving the early diagnosis performance of Alzheimer’s Disease lies in AD-related metrics. To present the results more clearly, metrics for AD and Non-AD are calculated separately in ADReSSo dataset and metrics for AD, MCI, and HC were calculated separately in NCMMSC2021 dataset. The confusion matrixes and the results of different groups are shown in Fig 3 and Table 6, respectively. From them, we can see that EDAMM achieves an accuracy of 93.0% on the NCMMSC2021(long) dataset, 77.0% on the NCMMSC2021(short) dataset, and 84.1% on ADReSSo in identifying AD. Compared to short audio, long audio demonstrates superior classification performance in AD, since short speech segments are derived from the segmentation of long speech, resulting in incomplete sentences and loss of semantic information. Therefore, long audio is more suitable for early Alzheimer’s Disease diagnosis tasks.

**Table 6:** Results of different groups on two datasets.

Dataset	Type	Accuracy(%)	Precision(%)	Recall(%)	F1(%)
NCMMSC2021(long)	AD	93.0±2.0	83.6±1.6	91.0±2.4	88.4±1.6
	MCI	83.3±1.3	88.1±1.1	83.3±1.4	86.0±1.2
	HC	94.3±1.9	98.9±1.1	95.6±2.1	97.0±1.4
NCMMSC2021(short)	AD	77.0±1.9	77.0±2.4	76.2±2.2	76.9±2.0
	MCI	81.7±1.2	80.0±2.0	81.1±1.9	80.6±1.9
	HC	91.0±1.6	90.2±2.7	90.3±2.1	90.7±1.9
ADReSSo	AD	84.1±1.7	88.4±1.6	84.0±1.6	85.6±1.9
	Non-AD	87.0±1.3	83.4±1.3	86.9±1.5	84.5±1.7

In conclusion, EDAMM proposed in this paper has shown validity in the task of early diagnosis of Alzheimer’s Disease.

### Ablation experiments

#### Ablation experiment for feature extraction module

To assess the effectiveness of the feature extraction module, three sets of experiments were conducted on different modalities in the NCMMSC2021 and ADReSSo datasets. The results are shown in Table 7. All the metrics are derived by calculating the average results of different groups.

**Table 7:** Ablation experiment for feature extraction module.

Dataset	Method number	Wav2Vec2.0	Word2Vec	TF-IDF	Accuracy (%)	Precision (%)	Recall (%)	F1(%)
NCMMSC2021(long)	a	√	×	×	84.3±2.4	84.3±2.5	84.6±2.0	84.3±2.4
	b	√	√	×	86.6±0.9	86.2±1.2	86.4±1.0	86.2±1.5
	c	√	√	√	87.8±1.1	87.2±1.3	87.6±1.1	87.4±1.1
NCMMSC2021(short)	a	√	×	×	78.2±2.4	78.4±2.0	78.0±1.9	78.0±1.9
	b	√	√	×	79.1±2.2	79.6±1.5	79.5±1.2	79.1±1.6
	c	√	√	√	82.0±2.5	81.6±2.2	81.6±2.4	81.6±2.4
ADReSSo	a	√	×	×	76.6±1.6	76.0±2.6	76.6±1.9	76.5±1.7
	b	√	√	×	78.4±1.2	79.1±1.3	78.6±1.5	78.3±1.2
	c	√	√	√	80.3±2.5	80.9±2.4	80.3±2.7	80.2±2.6

Method a: this method solely utilizes Wav2Vec 2.0 for the extraction of acoustic features, without integrating multimodal data fusion. This leads to the limitations of using single-modal features, resulting in comparatively poorer classification outcomes.

Method b: this technique employs multimodal data, building upon Method a. It incorporates Word2Vec to extract deep linguistic features from text, aiding in the comprehension of semantic information within transcribed texts.

Method c: this method further incorporates TF-IDF to extract traditional linguistic features from text, which assists in more effectively distinguishing key information within textual data. It has more holistic and multidimensional information, leading to the best classification results.

#### Ablation experiment for attention mechanism modules

To validate the effectiveness of self-attention mechanism and cross-modal attention mechanisms, three sets of experiments were conducted on the NCMMSC2021 and ADReSSo datasets. The detailed experimental results are provided in Table 8. All the metrics are derived by calculating the average results of different groups.

**Table 8:** Ablation experiment for attention mechanism modules.

Dataset	Method number	self-attention	cross-modal attention	Accuracy (%)	Precision (%)	Recall (%)	F1(%)
NCMMSC2021(long)	d	×	×	87.8±1.1	87.4±1.1	87.6±1.1	87.4±1.1
	e	√	×	88.0±2.1	87.6±1.9	87.9±2.2	87.6±1.9
	f	√	√	90.2±1.5	90.2±1.3	89.9±1.7	90.5±1.4
NCMMSC2021(short)	d	×	×	82.0±2.5	81.6±2.2	81.6±2.4	81.6±2.4
	e	√	×	82.3±2.3	82.1±2.0	81.9±1.9	82.3±2.2
	f	√	√	83.2±2.1	82.7±2.0	82.5±2.5	82.7±2.5
ADReSSo	d	×	×	80.3±2.5	80.9±2.4	80.3±2.7	80.2±2.6
	e	√	×	82.5±1.8	82.8±1.7	82.7±1.6	82.1±2.0
	f	√	√	85.5±1.3	85.9±1.2	85.4±1.5	85.0±1.8

Method d: acoustic and linguistic features were extracted using Wav2Vec2.0, TF-IDF and Word2Vec, followed by simple concatenation for early diagnosis. However, since the modal information was independent of each other, this method resulted in the poorest performance.

Method e: a self-attention mechanism was employed to extract fused features after concatenation, but the process introduced more noise, leading to suboptimal results.

Method f: cross-modal attention mechanisms were introduced to mitigate noise interference during multimodal fusion, enabling better obtaining useful information. By fully leveraging the correlations and complementarities among modal data, enhancing the representation capabilities of each modality, Method f yielded the best results. These experiments validate the effectiveness of the attention mechanism modules used in our study.

### Error analysis

In our study, we pay special attention to the internal validity of the model. To this end, we design a rigorous experimental process to reduce internal bias as much as possible. In addition, we thoroughly double-check the model training and evaluation process, ensuring the robustness and reliability of the results. However, there may still be some errors unnoticed, which requires us to be vigilant in later work.

To ensure the generalizability of the research results, we choose two different language datasets, NCMMSC2021 and ADReSSo, for evaluation. This multilingual approach helps us to test the stability and cross-lingual consistency of EDAMM, thus ensuring the external validity.

To evaluate the effectiveness of EDAMM, we use four evaluation metrics such as accuracy, precision, recall and F1 score, which are common-used in other researches on AD diagnosis. Thus, we believe there is little threat to construct validity.

In the future, in order to further improve EDAMM, we plan to use more datasets, explore new features and try other advanced techniques. In addition, we will cooperate with clinics to promote the early diagnosis of AD.

## Conclusion

In this paper, we propose a novel approach EDAMM for early diagnosis of Alzheimer’s Disease. EDAMM helps the early diagnosis of AD in three ways, i.e., non-invasive feature extraction, attention mechanism and effective diagnosis.

Non-invasive feature extraction: EDAMM employs three feature extraction methods, Wav2Vec2.0, TF-IDF, and Word2Vec, to extract acoustic and linguistic features. These features can be extracted by a non-invasive and cost-effective way. In addition, acoustic features can capture subtle changes in patient speech, while linguistic features can reflect anomalies in language use. The complementarity of these features is helpful for a comprehensive analysis of patient symptoms.

Attention mechanism: EDAMM leverages both the self-attention mechanism and the cross-modal attention mechanism to process the acoustic and linguistic features. These attention mechanisms help to fully utilize the information in multimodal data.

Effective diagnosis: EDAMM shows good performance in the early diagnosis of AD. Specifically, Experimental results demonstrate that EDAMM achieves an accuracy of 91.6% in diagnosing AD on the Chinese dataset NCMMSC2021, and 86.7% on the English dataset ADReSSo.

In the future, we plan to further improve the performance of Alzheimer’s Disease diagnosis by introducing additional relevant features and better diagnosis technologies. We also plan to perform experiments on more datasets to reduce the threats to external validity. In addition, we will cooperate with clinics to promote the early diagnosis of AD.
